# Fetal sex and the relative reactivity of human umbilical vein and arteries are key determinants in potential beneficial effects of phosphodiesterase inhibitors

**DOI:** 10.1152/japplphysiol.00540.2023

**Published:** 2024-05-02

**Authors:** Anne-Christine Peyter, Manon Beaumann, Flavien Delhaes, Sébastien Joye, Steeve Menétrey, David Baud, Jean-François Tolsa

**Affiliations:** ^1^Neonatal Research Laboratory, Department Woman-Mother-Child, Lausanne University Hospital and University of Lausanne, Lausanne, Switzerland; ^2^Clinic of Neonatology, Department Woman-Mother-Child, Lausanne University Hospital and University of Lausanne, Lausanne, Switzerland; ^3^Clinic of Gynecology and Obstetrics, Department Woman-Mother-Child, Lausanne University Hospital and University of Lausanne, Lausanne, Switzerland

**Keywords:** cyclic nucleotide phosphodiesterase, intrauterine growth restriction, human umbilical artery, human umbilical vein, nitric oxide

## Abstract

Intrauterine growth restriction (IUGR) is a common complication of pregnancy. We previously demonstrated that IUGR is associated with an impaired nitric oxide (NO)-induced relaxation in the human umbilical vein (HUV) of growth-restricted females compared to appropriate for gestational age (AGA) newborns. We found that phosphodiesterase (PDE) inhibition improved NO-induced relaxation in HUV, suggesting that PDEs could represent promising targets for therapeutic intervention. This study aimed to investigate the effects of PDE inhibition on human umbilical arteries (HUAs) compared to HUV. Umbilical vessels were collected in IUGR and AGA term newborns. NO-induced relaxation was studied using isolated vessel tension experiments in the presence or absence of the nonspecific PDE inhibitor 3-isobutyl-1-methylxanthine (IBMX). PDE1B, PDE1C, PDE3A, PDE4B, and PDE5A were investigated by Western blot. NO-induced vasodilation was similar between IUGR and AGA HUAs. In HUAs precontracted with serotonin, IBMX enhanced NO-induced relaxation only in IUGR females, whereas in HUV IBMX increased NO-induced relaxation in all groups except IUGR males. In umbilical vessels preconstricted with the thromboxane A2 analog U46619, IBMX improved NO-induced relaxation in all groups to a greater extent in HUV than HUAs. However, the PDE protein content was higher in HUAs than HUV in all study groups. Therefore, the effects of PDE inhibition depend on the presence of IUGR, fetal sex, vessel type, and vasoconstrictors implicated. Despite a higher PDE protein content, HUAs are less sensitive to IBMX than HUV, which could lead to adverse effects of PDE inhibition in vivo by impairment of the fetoplacental hemodynamics.

**NEW & NOTEWORTHY** The effects of phosphodiesterase inhibition on the umbilical circulation depend on the presence of intrauterine growth restriction, the fetal sex, vessel type, and vasoconstrictors implicated. The human umbilical vascular tone regulation is complex and depends on the amount and activity of specific proteins but also probably on the subcellular organization mediating protein interactions. Therefore, therapeutic interventions using phosphodiesterase inhibitors to improve the placental-fetal circulation should consider fetal sex and both umbilical vein and artery reactivity.

## Graphical abstract

**Figure:**
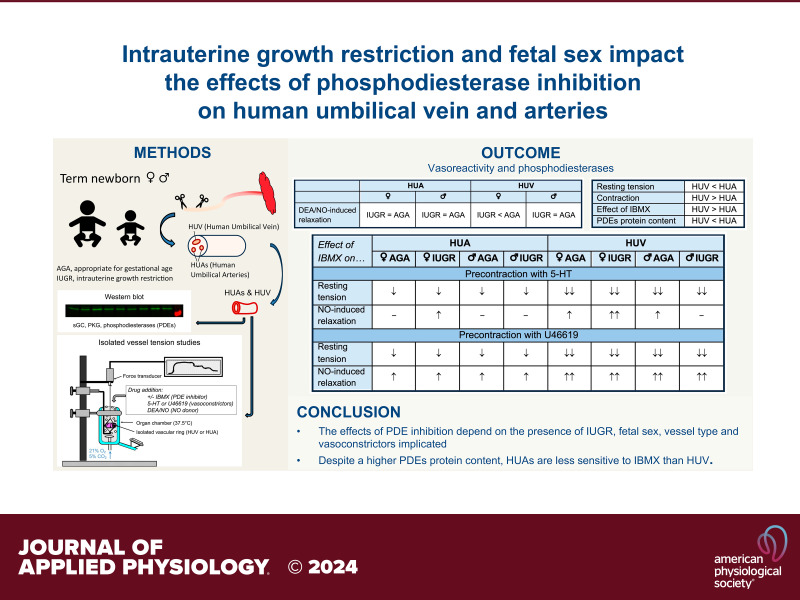

## INTRODUCTION

Intrauterine growth restriction (IUGR) is a common complication of pregnancy, associated with major perinatal mortality and morbidity, as well as with an increased risk of developing cardiovascular and metabolic diseases in adulthood (1). This pathology is a public health concern, linked to high healthcare costs worldwide. Despite many animal and human studies assessing substances that have the potential to improve fetal growth (2), there is currently no effective method to prevent or treat IUGR and limit its short- and long-term adverse consequences. IUGR is characterized by an abnormal fetal growth slowing below expectations, resulting in a small body weight at birth compared to newborns with harmonious fetal growth at the same gestational age [appropriate for gestational age (AGA)]. Outside increased risks of prematurity, the long-term adverse effects of IUGR are mainly related to reprogramming processes aiming to improve fetal survival by sparing the main organs (3). Despite the identification of some risk factors, the mechanisms contributing to the development of IUGR remain poorly understood, while diagnosis and management of this pathology are still a challenge (4).

We previously demonstrated that IUGR is associated with structural and functional alterations in the umbilical cord of human term newborns (5, 6). In particular, we found a reduced umbilical cord diameter, as well as a decreased total cross-sectional area and smooth muscle area in the human umbilical vein (HUV) of growth-restricted compared to AGA newborns, but no significant morphometrical difference in human umbilical arteries (HUAs) between IUGR and AGA newborns (6). We also showed that IUGR is associated with sex-specific alterations in the nitric oxide (NO)/cyclic GMP (cGMP)-mediated relaxing pathway in the HUV (5, 6): we observed a reduced relaxant response to the NO donor 2-(*N*,*N*-diethylamino)-diazenolate-2-oxide (DEA/NO) in the HUV of growth-restricted females compared to AGA newborns, despite a significant increase in soluble guanylyl cyclase (sGC) protein content and activity and some increase in cGMP-dependent protein kinase (PKG) protein amount compared to AGA HUV; in males, however, no significant difference was found between AGA and IUGR HUV. Because the HUV is the only vessel carrying the blood from the placenta to the fetus, it is likely that such alterations could contribute to the development of IUGR. Finally, we found a beneficial effect of phosphodiesterase (PDE) inhibition by the nonspecific PDE inhibitor 3-isobutyl-1-methylxanthine (IBMX) on the NO-induced relaxation in HUV (5), which could represent an interesting therapeutic approach to improve placental-fetal blood flow.

However, an international randomized clinical trial using sildenafil versus placebo in pregnancies with severe early-onset fetal growth restriction, the STRIDER trial, was suddenly interrupted due to a lack of benefit and unexpected postnatal deaths in the treated group from the Netherlands (7). No beneficial effect of the maternal treatment with the PDE5 inhibitor sildenafil was found on fetal growth, perinatal mortality, or major neonatal morbidity, as confirmed by the United Kingdom, New Zealand, and Australian STRIDER trials (8). Moreover, the Dutch STRIDER trial reported an increased rate of pulmonary hypertension in live born infants in the sildenafil group (18.8%) versus the placebo group (5.1%) (7). The pathophysiological mechanism associated with this increase in persistent pulmonary hypertension of the newborn (PPHN) after prolonged maternal treatment with sildenafil remains unclear, but the authors hypothesized post hoc that pulmonary hypertension could result from a “rebound” vasoconstriction following discontinuation of the treatment (9).

These findings, coupled with our previous results, support the need to further investigate the contribution of PDEs to the regulation of the human umbilical vascular tone.

Overall, our project aims to better understand the regulation of the human umbilical circulation. The vasoreactivity of HUAs was therefore investigated in the same conditions we previously published for HUV. Isolated vessel tension experiments in organ bath were performed simultaneously in HUV and HUAs isolated from the same patient, enabling direct comparison between the two vessels. Studying the effects of PDE inhibition in HUAs aimed to verify that such a promising intervention to improve blood flow in HUV would not have adverse effects on HUAs. Indeed, the placental-fetal circulation is a “closed” system, so it is likely that any intervention on one part of it would have overall effects on the whole system.

## MATERIALS AND METHODS

### Biological Samples Collection

The present study was a direct continuation of our previous works (5, 6), approved by the ethical committee of the Faculty of Biology and Medicine of the University of Lausanne (Protocol No. 134/08). Data presented in this manuscript were obtained using biological samples from the same patients already included in our previous study (5). The vasoreactivity of HUV and HUAs isolated from the same patient was assessed simultaneously during each organ bath experiment, the results of which are presented in this report and in our previous publication (5). Umbilical cords were collected in 388 newborns delivered at the Maternity of the University Hospital CHUV in Lausanne (Switzerland) between July 2009 and January 2017.

### Patients: Inclusion/Exclusion Criteria and Classification

Inclusion criteria were term (≥37 accomplished weeks of gestation) singleton pregnancies. Exclusion criteria were newborns with a birth weight (BW) above the 90th percentile (P90), fetal abnormalities, genetic syndromes, single HUA, mothers presenting with HIV; hepatitis A, B, or C; and preeclampsia.

Newborns were dichotomized into two categories, based on their body weight at birth: “IUGR” and “AGA.” Samples were assigned to the “AGA” group when BW was between the 10th percentile (P10) and P90 and to the “IUGR” group when BW was below P10. The percentile classification was determined using a growth percentile calculator, which consists of an Excel file using a formula based on data manually extracted from growth charts endorsed by the Swiss Society of Pediatrics (10). Male and female newborns were studied separately. However, since prenatal growth follow-up or other parameters like ultrasound/Doppler measurements were not available for all patients, our subjects were retroactively reclassified according to the criteria of the “Consensus Based Definition of Growth Restriction in the Newborn” published in *The Journal of Pediatrics* (2018) (11). This allowed a more rigorous postnatal classification of our patients. Subjects who did not reach these criteria were excluded from the initial groups.

### Samples Collection

The proximal part of the umbilical cord was collected at delivery and used within 24 h. A 10- to 15-cm segment was cut as close as possible to the fetus and kept, until dissection, at 4°C in modified Krebs-Ringer solution (118.3 mM NaCl, 4.7 mM KCl, 2.5 mM CaCl_2_, 1.2 mM MgSO_4_, 1.2 mM KH_2_PO_4_, 25.0 mM NaHCO_3_, and 11.1 mM glucose), previously bubbled with 21% O_2_-5% CO_2_ during ∼30 min (6).

### Isolated Vessel Tension Experiments

HUAs reactivity was investigated as previously described for HUV (5). Briefly, both HUAs were dissected, cut into small rings (4–5 mm in length, 2 rings per artery), suspended between two stirrups (diameter of 0.20 mm) passed through the lumen into vertical organ chambers filled with 10 mL modified Krebs-Ringer solution aerated with 21% O_2_-5% CO_2_, and maintained at 37.5°C. Isometric tension was measured using a strain gauge connected to a bridge amplifier (PowerLab, ADInstruments). A 2-g stretch tension was applied to each vascular ring, followed by 20-min equilibration before washing. Stretch/equilibration/wash steps were processed four times to get the vessel rings to their optimal resting tension. After equilibration, the vessels were challenged with 100 mM KCl to test their viability. After washing and equilibration, indomethacin (10^−5^ M) and *N*^G^-nitro-l-arginine (NLA; 10^−4^ M) were added to exclude possible interference of endogenous prostanoids and NO. NLA 10^−4^ M was shown to inhibit endothelium-dependent relaxation in human umbilical vessels (12, 13); this was confirmed by preliminary data in our experimental conditions (data not shown). IBMX (10^−4^ M), a nonspecific inhibitor of PDEs, was also added in some of these experiments to investigate the contribution of PDEs in NO-induced relaxation. Vascular rings were then precontracted with serotonin (5-HT) or U46619, an analog of thromboxane A2 (TxA2), and finally relaxed with cumulative doses of the NO donor 2-(*N*,*N*-diethylamino)-diazenolate-2-oxide (DEA/NO). Preliminary experiments showed that 10^−5^ M 5-HT or 10^−6^ M U46619 induced a sustained contraction in HUV and HUAs (6). Table 1 presents the pharmacological agents and concentrations used in these experiments. The resting tension (RT) was determined as the lowest tension achieved by each vascular ring during the entire isolated vessel tension experiment after the stretch/equilibration/wash steps. The residual tension (RDT) corresponded to the tension (after subtraction of the corresponding RT) obtained following contraction with 5-HT or U46619, measured at the time the first dose of DEA/NO was added. Change in tension induced by DEA/NO was expressed as a percentage of the initial contraction induced by the vasoconstrictor (RDT).

**Table 1.** Pharmacological agents used in the isolated vessel tension experiments

Pharmacological Agent	Effect	Concentration in Organ Bath
KCl (potassium chloride)	Vasoconstrictor	100 mM
5-HT (serotonin or 5-hydroxytryptamine)	Vasoconstrictor	10^−5^ M
U46619 (thromboxane A2 analog)	Vasoconstrictor	10^−6^ M
DEA/NO [2-(*N*, *N*-diethylamino)-diazenolate-2-oxide]	NO donor, vasodilator	10^−8^ to 10^−4^ M
IBMX (3-isobutyl-1-methylxanthine)	Nonspecific PDE inhibitor	10^−4^ M

NO, nitric oxide; PDE, phosphodiesterase.

For each dose-response curve to DEA/NO, area under the curve (AUC), half-maximal effective concentration (EC_50_), and maximum effect (E_max_) were calculated using Prism 9.5 [GraphPad Prism; Research Resource Identifier (RRID):SCR_002798] from the relaxation curve obtained in umbilical vessels from each patient.

The dose-response curves obtained in HUV collected from the same patients as HUAs used in the present study were previously published in Ref. 5. However, they were presented with an AUC calculated from the “global” dose-response to DEA/NO (instead of individual dose-response curves as for HUAs), and the associated EC_50_ and E_max_ had not yet been published. Therefore, the corresponding AUCs were recalculated from the individual dose-response curves obtained for each patient to allow direct comparison with the AUC calculated in HUAs collected from the same patients.

### Western Blot

The relative amounts of sGC, PKG, PDE1B, PDE1C, PDE3A, PDE4B, and PDE5A in umbilical vessels homogenates were investigated by Western blot as previously described (5). However, in contrast to our previous study, the specific protein content was normalized to the total protein amount using Ponceau S staining (instead of actinin content), which is now recognized as a more reliable approach (14). Flash-frozen HUA or HUV segments were crushed in a cryogenic mortar, homogenized in lysis buffer {50 mM HEPES, 1 mM EDTA, 1 mM EGTA, 10% glycerol, 1 mM DTT, 5 µg/ml pepstatin, 3 µg/ml aprotinin, 10 µg/ml leupeptin, 0.1 mM 4-(2-aminoethyl) benzenesulfonyl fluoride hydrochloride (AEBSF), 1 mM sodium vanadate, 50 mM sodium fluoride, and 20 mM 3-[(3-cholamidopropyl)dimethylammonio]-1-propanesulfonate (CHAPS)}, and centrifuged for 10 min at 3,000 *g* at 4°C. Supernatant protein concentration was quantified using a BCA protein assay kit (Pierce, cat. no. 23227). Samples were diluted in Laemmli buffer and heated for 5 min at 95°C before loading on a 7.5% polyacrylamide gel. Proteins were separated by SDS-PAGE (1 h 30 min, 0.05 A) and transferred to a nitrocellulose membrane (overnight, 30 V, 4°C). A Ponceau S total protein staining (Sigma-Aldrich, cat. no. P7170) was performed to visualize proteins and the stained membrane was scanned, using an office scanner, to allow further normalization. After the staining was removed, membranes were blocked in casein solution (Vector, cat. no. SP-5020) (30 min, at room temperature) and immunoblotted (overnight, 4°C) using specific antibodies against sGC (1:2,000, Abcam, cat. no. ab50358, RRID:AB_880325), PKG (1:1,000, Enzo Life Sciences, cat. no. ADI-KAP-PK005-D, RRID:AB_2039483), PDE1B (1:2,000, OriGene, cat. no. TA503319, RRID:AB_11125416), PDE1C (1:250, GeneTex, cat. no. GTX14602, RRID:AB_368824), PDE3A (1:1,000, GeneTex, cat. no. GTX112305, RRID:AB_11168131), PDE4B (1:1,000, GeneTex, cat. no. GTX113798, RRID:AB_11173431), or PDE5A (1:500, Santa Cruz Biotechnology, cat. no. sc-32884, RRID:AB_2161266), diluted in blocking solution containing 0.1% Tween. Blots were then incubated with an anti-mouse or anti-rabbit antibody (IRDye, LI-COR Biosciences cat. no. 926–32212, RRID:AB_621847 and cat. no. 926–68073, RRID:AB_10954442) diluted 1:10,000 in casein solution (1 h, at room temperature). Target proteins were visualized using an Odyssey Infrared Imaging System (LICOR). The amount of each specific protein was quantified using the image processing software ImageJ (RRID:SCR_003070) and normalized to the corresponding Ponceau S total protein staining.

For each experimental group, samples from 40 patients were randomly distributed into four pools, to limit the influence of individual variability on the results. To allow comparison between the different membranes and groups, the specific protein content measured in each pool was reported to the amount measured in a “standard” sample included on each membrane. The latter was constituted of proteins extracted from six additional patients (3 AGA males and 3 AGA females), which were not included in the experimental pools. At least three Western blots were performed for each protein. The relative content calculated for each pool corresponded to the mean of the relative amounts determined in the greater than or equal to three blots.

### PDE Activity in HUV Homogenates

The PDE activity was quantified in HUV homogenates using a cyclic nucleotide phosphodiesterase assay kit (Enzo Life Sciences, cat. no. BML-AK800-0001) according to the manufacturer’s instructions, as previously published (5). Briefly, HUV homogenates were prepared using a tissue grinder after mixing HUV segments, previously flash-frozen and crushed in a cryogenic mortar, with assay buffer supplemented with 20 mM CHAPS. They were then sonicated and centrifuged for 10 min at 3,000 *g* at 4°C. A buffer exchange was performed using Pierce Protein Concentrators PES, 3 K molecular weight cut-off (Life Technologies, cat. no. 88512), 0.5 mL, to remove excess free phosphate, which interferes with the BIOMOL GREEN reagent of the kit. Enzymatic reaction was performed during 20 min at 30°C in assay buffer supplemented with 0.5 mM MgCl_2_, 0.2 mM CaCl_2,_ and 2 mM calmodulin. cGMP or cAMP degradation was tested in the absence or presence of IBMX 10^−4^ M. For each sample, total and IBMX-sensitive PDE activity was normalized by the corresponding protein content, previously quantified using the BCA protein assay kit, and expressed as nmol cGMP or cAMP/mg protein/min.

### Data Analyses

Statistical analyses were performed using the software Prism 9.5 (GraphPad Prism; RRID:SCR_002798). The dose-response curves to DEA/NO were analyzed by two-way ANOVA with Tukey’s multicomparison test, except for direct comparisons between HUV and HUAs or between vascular rings precontracted with 5-HT or U46619, where two-way ANOVA with matched-paired values and Sidák’s multicomparisons test was used. For the other parameters (AUC, RT, RDT, EC_50_, E_max_, contraction induced by KCl, demographic data, and relative protein content), the data obtained in two study groups were compared using a Mann-Whitney test, except for direct comparisons between HUV and HUAs or between vascular rings precontracted with 5-HT or U46619, where a Wilcoxon matched-pairs sign rank was performed. Statistical analyses for direct comparisons between HUV and HUAs were performed on matched-paired data because HUV and HUAs were collected from the same patient and their vasoreactivity was studied in parallel during each organ bath experiment; similarly, the vasorelaxant responses after precontraction with 5-HT or U46619 were tested simultaneously on vascular rings isolated from the same vessel. Finally, statistical analyses of PDE activity in HUV were performed using Brown-Forsythe and Welch ANOVA tests. The differences were considered statistically significant when *P* < 0.05.

## RESULTS

### Demographic Data

The maternal and neonatal characteristics were significantly different between AGA and IUGR groups, except for maternal age and gestational age at birth, as presented in Table 2. Body weight, length, head circumference, ponderal index, placental weight, and umbilical cord diameter were significantly reduced in IUGR newborns, whereas the body-to-placental weight ratio and cord diameter-to-placental weight ratio were increased.

**Table 2.** Demographic data related to patients included in the present study

	AGA	IUGR	*P* Value
Females			
Number of patients included	122	79	
Gestational age, wk	39.6 ± 1.0	39.3 ± 1.0	0.1402
Birth weight, g	3,327 ± 308	2,549 ± 282*	<0.0001
Length, cm	49.1 ± 1.6	46.4 ± 2.1*	<0.0001
Head circumference, cm	34.4 ± 1.2	32.7 ± 1.1*	<0.0001
Ponderal index, g/cm^3^	2.82 ± 0.22	2.55 ± 0.26*	<0.0001
Placental weight, g	622 ± 116	425 ± 82*	<0.0001
Body-to-placental weight ratio	5.48 ± 0.88	6.14 ± 1.03*	<0.0001
Umbilical cord diameter, mm	12.3 ± 2.6	10.6 ± 2.0*	<0.0001
Cord diameter-to-placental weight ratio	0.020 ± 0.006	0.026 ± 0.007*	<0.0001
Maternal age, yr	32.8 ± 9.1	31.6 ± 5.3	0.3360
Males			
Number of patients included	121	66	
Gestational age, wk	39.4 ± 1.0	39.3 ± 1.1	0.9027
Birth weight, g	3,414 ± 304†	2,614 ± 287*	<0.0001
Length, cm	49.7 ± 1.7†	46.7 ± 1.8*	<0.0001
Head circumference, cm	35.1 ± 1.0†	33.1 ± 1.2*†	<0.0001
Ponderal index, g/cm^3^	2.78 ± 0.22	2.56 ± 0.20*	<0.0001
Placental weight, g	613 ± 114	441 ± 96*	<0.0001
Body-to-placental weight ratio	5.72 ± 0.91	6.15 ± 1.17*	0.0055
Umbilical cord diameter, mm	13.1 ± 3.0	11.6 ± 2.5*†	0.0001
Cord diameter-to-placental weight ratio	0.022 ± 0.007	0.027 ± 0.007*	<0.0001
Maternal age, yr	33.0 ± 5.2	31.4 ± 5.8	0.1059

Data are expressed as means ± SD. Ponderal index was calculated as [100 × birth weight/length^3^] (g/cm^3^). *P* values presented are related to comparison between appropriate for gestational age (AGA) and intrauterine growth-restricted (IUGR) groups. All parameters were analyzed using a Mann-Whitney test. *Significant difference between AGA and IUGR newborns. †Significant difference between males and females.

### Vasoreactivity

#### NO-induced relaxation in HUAs.

**Figure 1.**
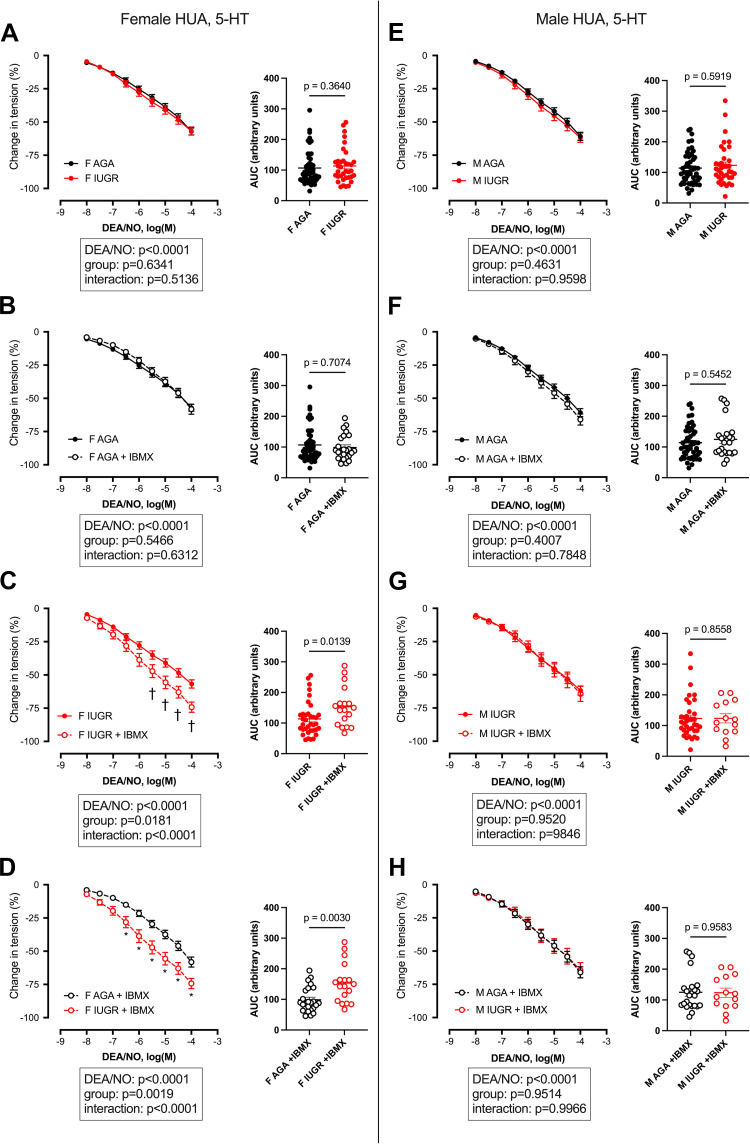
Relaxation induced by cumulative doses of the nitric oxide donor 2-(*N*,*N*-diethylamino)-diazenolate-2-oxide (DEA/NO) on isolated umbilical arteries precontracted with 5-HT in the absence or presence of IBMX. AGA, appropriate for gestational age; F, females; IUGR, intrauterine growth restriction; M, males. Cumulative doses of DEA/NO were applied to human umbilical arteries (HUAs), isolated from newborn females (*A*–*D*) or males (*E*–*H*), precontracted with 5-HT 10^−5^ M in the absence or presence of IBMX 10^−4^ M. Data are expressed as means ± SE of the percentage of change in tension induced by the vasodilator (*n* = 18–51 for females; *n* = 14–50 for males). Data were analyzed by two-way ANOVA with Tukey’s multicomparison test (results are shown at *bottom*). *Significant difference between AGA and IUGR. †Significant difference between absence and presence of IBMX (*P* < 0.05). For each study group, the corresponding area under the curve (AUC) was calculated from the individual dose-response curves obtained for each patient. Results are expressed as individual values, with line at means ± SE (*n* = 18–51 for females; *n* = 14–50 for males). *P* values were obtained using a Mann-Whitney test to compare both groups.

**Figure 2.**
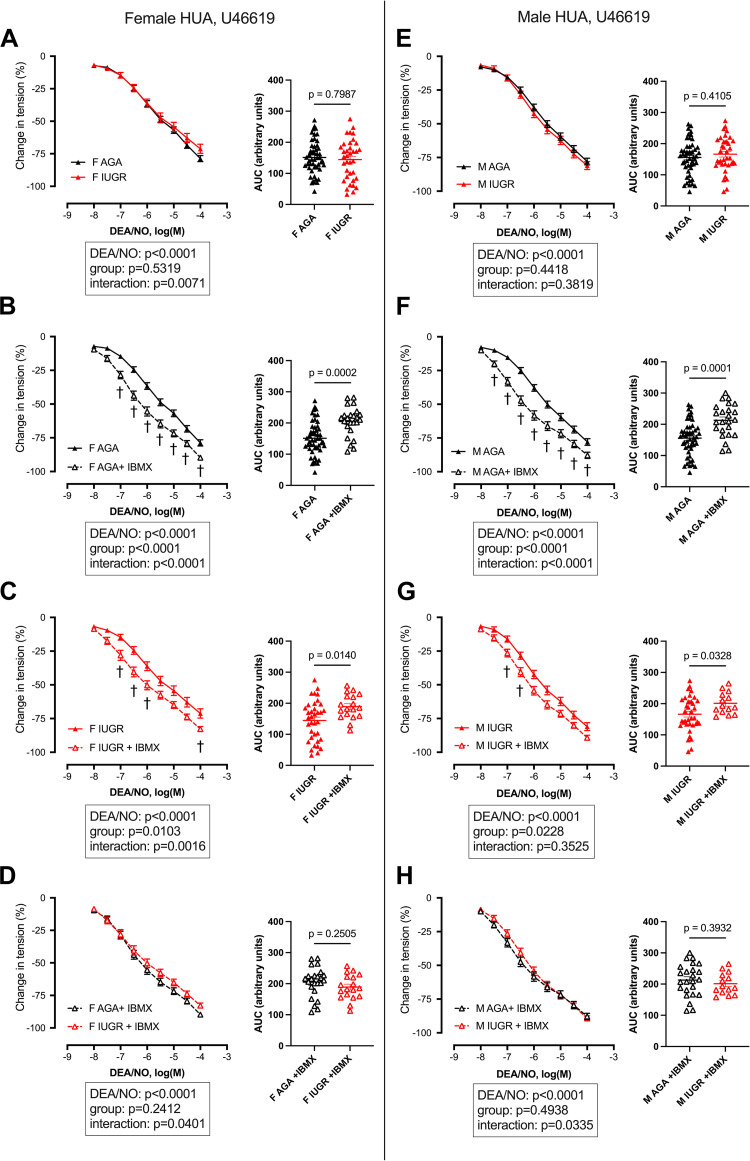
Relaxation induced by cumulative doses of the nitric oxide donor 2-(*N*,*N*-diethylamino)-diazenolate-2-oxide (DEA/NO) on isolated umbilical arteries precontracted with U46619 in the absence or presence of IBMX. AGA, appropriate for gestational age; F, females; IUGR, intrauterine growth restriction; M, males. Cumulative doses of DEA/NO were applied to human umbilical arteries (HUAs) isolated from newborn females (*A*–*D*) or males (*E*–*H*), precontracted with U46619 10^−6^ M in the absence or presence of IBMX 10^−4^ M. Data are expressed as means ± SE of the percentage of change in tension induced by the vasodilator (*n* = 18–51 for females; *n* = 14–50 for males). Data were analyzed by two-way ANOVA with Tukey’s multicomparison test (results are shown at *bottom*). †Significant difference between absence and presence of IBMX (*P* < 0.05); no significant difference was found between IUGR and AGA neonates. For each study group, the corresponding area under the curve (AUC) was calculated from the individual dose-response curves obtained for each patient. Results are expressed as individual values, with line at means ± SE (*n* = 18–51 for females; *n* = 14–50 for males). *P* values were obtained using a Mann-Whitney test to compare both groups.

Cumulative doses of DEA/NO induced, in all study groups, a dose-dependent relaxation in HUAs preconstricted with 5-HT (Fig. 1) or U46619 (Fig. 2). No significant difference was observed, with both vasoconstrictors, between AGA and IUGR newborns, either in females (Fig. 1*A* and 2*A*) or in males (Figs. 1*E* and 2*E*). The corresponding EC_50_ and Emax are presented in Supplemental Tables S1 and S2, respectively.

#### Effects of PDE inhibition in HUAs.

Dose response to DEA/NO was then investigated in HUAs preincubated with the nonspecific PDE inhibitor IBMX (10^−4^ M). In HUAs precontracted with 5-HT, the presence of IBMX did not influence the relaxant response to DEA/NO in AGA females (Fig. 1*B*) or males (Fig. 1*F*) or in IUGR males (Fig. 1, *G*–*H*). In IUGR females, however, IBMX significantly improved DEA/NO-induced relaxation, with a significant increase in AUC (+35%) compared to IUGR female HUAs without IBMX (Fig. 1*C*). Pretreatment of HUAs with IBMX therefore resulted in a significantly better relaxant response to DEA/NO in IUGR than AGA females, with a significantly higher AUC (+55%) (Fig. 1*D*).

In HUAs preconstricted with U46619, the DEA/NO-induced relaxation was significantly improved by IBMX in all groups (Fig. 2, *B*, *C*, *F* and *G*), with a significant increase in AUC (+35% in AGA females, +37% in AGA males, +31% in IUGR females, and +21% in IUGR males). The resulting dose-response curves obtained after preincubation with IBMX were similar between AGA and IUGR females (Fig. 2*D*) or between AGA and IUGR males (Fig. 2*H*).

#### Comparison between 5-HT and U46619.

**Figure 3.**
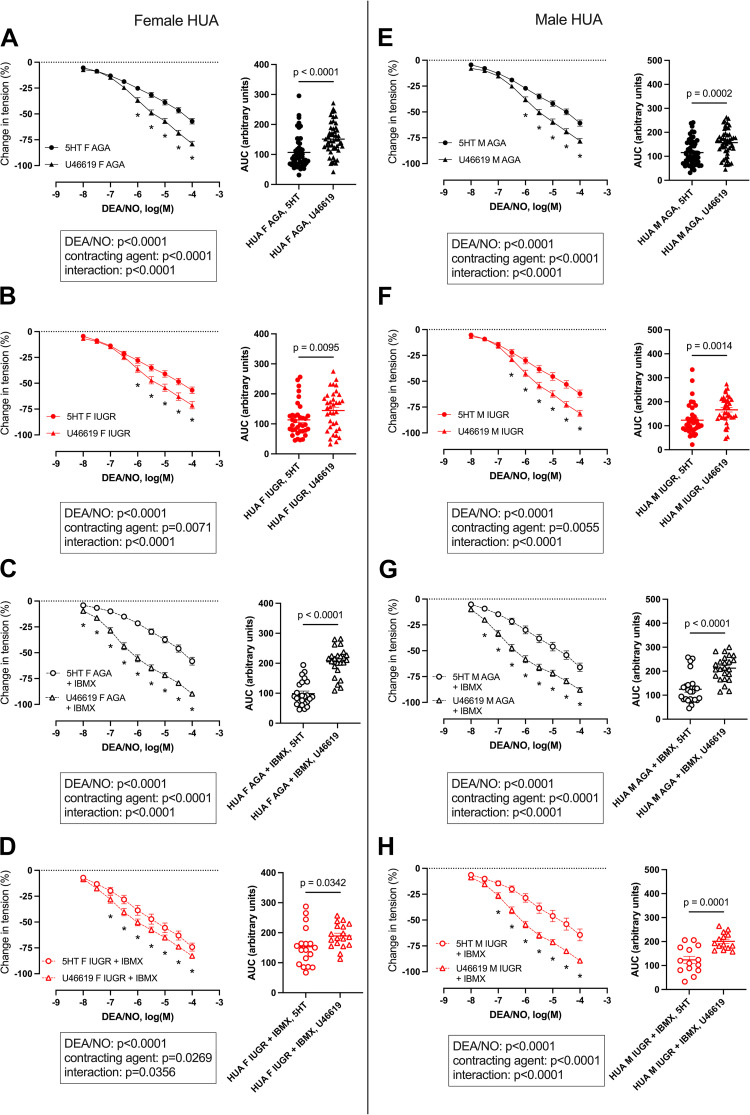
Relaxation induced by cumulative doses of the nitric oxide donor 2-(*N*,*N*-diethylamino)-diazenolate-2-oxide (DEA/NO) on isolated umbilical arteries precontracted with 5-HT or U46619 in the absence or presence of IBMX. AGA, appropriate for gestational age; F, females; M, males; IUGR, intrauterine growth restriction. Cumulative doses of DEA/NO were applied to human umbilical arteries (HUAs) isolated from newborn females (*A*–*D*) or males (*E*–*H*) precontracted with 5-HT 10^−5^ M or U46619 10^−6^ M in the absence or presence of IBMX 10^−4^ M. Data are expressed as means ± SE of the percentage of change in tension induced by the vasodilator (*n* = 18–51 for females; *n* = 14–50 for males). Data were analyzed by two-way ANOVA with matched-paired values and Sidák’s multicomparison test (results are shown at *bottom*) to compare DEA/NO-induced relaxation between HUAs isolated from the same patient precontracted with 5-HT or U46619. *Significant difference between 5-HT and U46619 (*P* < 0.05). For each study group, the corresponding area under the curve (AUC) was calculated from the individual dose-response curves obtained for each patient. Results are expressed as individual values, with line at means ± SE (*n* = 18–51 for females; *n* = 14–50 for males). *P* values were obtained using a Wilcoxon matched-pairs sign-ranked test to compare 5-HT and U46619 in HUAs isolated from the same patient.

Figure 3 shows a direct comparison between dose-response curves to DEA/NO obtained in HUAs precontracted with 5-HT or U46619. A similar comparison was performed for HUV precontracted with 5-HT or U46619 (Supplemental Fig. S1). In all groups and conditions, DEA/NO-induced relaxation was greater in umbilical vascular rings precontracted using U46619 than 5-HT, with significantly higher AUC.

In the absence of IBMX, the resting tension (RT) achieved in HUAs was similar with both vasoconstrictors, in all study groups (Table 3). After preincubation with IBMX, RT was significantly reduced in HUAs contracted with U46619 compared to 5-HT in AGA females and males but was similar with both vasoconstrictors in IUGR females and males (Table 3). In HUV, RT was similar with both vasoconstrictors in all groups except in IUGR females, where RT was significantly increased in HUV contracted with U46619 compared to 5-HT, independently of IBMX (Table 3).

**Table 3.** Resting tension achieved in vascular rings preconstricted with 5HT or U46619 and relaxed with DEA/NO

	F AGA	F IUGR	M AGA	M IUGR	F AGA	F IUGR	M AGA	M IUGR
	–IBMX				+IBMX			
5-HT								
HUA								
* n* (patients)	51	36	50	37	24	18	24	14
RT, g	0.65 ± 0.04	0.59 ± 0.03	0.66 ± 0.04	0.73 ± 0.05 †	0.34 ± 0.03	0.34 ± 0.04	0.41 ± 0.03	0.49 ± 0.05†
* P* value IUGR vs. AGA		0.7364		0.2166		0.7484		0.1981
*P* value +IBMX vs. −IBMX					<0.0001*** (−48%)	<0.0001*** (−42%)	<0.0001*** (−38%)	0.0035** (−33%)
HUV								
* n* (patients)	51	36	50	37	24	18	24	14
RT, g	0.20 ± 0.02	0.25 ± 0.04	0.24 ± 0.03	0.34 ± 0.05	0.05 ± 0.02	0.08 ± 0.03	0.09 ± 0.02	0.14 ± 0.04
* P* value IUGR vs. AGA		0.5044		0.0771		0.3403		0.3180
* P* value +IBMX vs. −IBMX					<0.000*** (−75%)	0.001*** (−68%)	0.0001*** (−63%)	0.0071** (−59%)
* P* value HUV vs. HUA	<0.0001***	<0.0001***	<0.0001***	<0.0001***	<0.0001***	<0.0001***	<0.0001***	0.0001***
U46619								
HUA								
* n* (patients)	51	36	50	37	24	18	24	14
RT, g	0.66 ± 0.04	0.65 ± 0.04	0.69 ± 0.05	0.64 ± 0.04	0.28 ± 0.02	0.35 ± 0.04	0.34 ± 0.02	0.44 ± 0.05
* P* value IUGR vs. AGA		0.8960		0.7829		0.2475		0.0566
* P* value +IBMX vs. −IBMX					<0.0001*** (−58%)	<0.0001*** (−46%)	<0.0001*** (−51%)	0.0054** (−31%)
* P* value HUA U46619 vs. 5-HT	0.9020	0.0582	0.4025	0.1532	0.0151* (−18%)	0.6927	0.0448* (−17%)	0.2474
HUV								
* n* (patients)	50	36	50	37	24	18	24	14
* *RT, g	0.22 ± 0.02	0.30 ± 0.04	0.26 ± 0.03	0.32 ± 0.04	0.06 ± 0.02	0.12 ± 0.03	0.07 ± 0.01	0.16 ± 0.05
* P* value IUGR vs. AGA		0.2858		0.5917		0.0717		0.4403
* P* value +IBMX vs. −IBMX					<0.0001*** (−73%)	0.0004*** (−60%)	<0.0001*** (−73%)	0.0084** (−50%)
* P* value HUV vs. HUA	<0.0001***	<0.0001***	<0.0001***	<0.0001***	<0.0001***	0.0002***	<0.0001***	0.0017**
* P* value HUV U46619 vs. 5-HT	0.1830	0.0143* (+20%)	0.1088	0.9839	0.5272	0.0144* (+50%)	0.8607	0.4919

Data are expressed as means ± SE. Values in parentheses correspond to the percentage of change between both groups, when difference was significant. The resting tension (RT) was determined as the lowest tension reached by each vascular ring during the isolated vessel tension experiment after the equilibration steps. *P* values resulted from statistical analyses using a Mann-Whitney test to compare intrauterine growth restriction (IUGR) and appropriate for gestational age (AGA) or RT in vascular rings treated or not with IBMX; a Wilcoxon matched-pairs sign rank test was used to compare human umbilical artery (HUA) and human umbilical vein (HUV) or vessels treated with U46619 or 5-HT. DEA/NO, 2-(*N*,*N*-diethylamino)-diazenolate-2-oxide. †Significant difference between males (M) and females (F) using a Mann-Whitney test. *P < 0.05, **P < 0.001, ***P < 0.0001, significant values.

#### Comparison between HUAs and HUV.

Supplemental Figs. S2 and S3 show a direct comparison between dose-responses to DEA/NO assessed in HUAs or HUV preconstricted with 5-HT (Supplemental Fig. S2) or U46619 (Supplemental Fig. S3). In female umbilical vessels precontracted with 5-HT, HUAs and HUV from AGA females showed quite similar responses to DEA/NO (Supplemental Fig. S2*A*). In IUGR females, the DEA/NO-induced relaxation was greater in HUAs than HUV, with a significantly higher AUC (+36%) (Supplemental Fig. S2*B*). In AGA female vessels preincubated with IBMX, DEA/NO-induced relaxation was significantly stronger in HUV than HUAs, with a significantly higher AUC (+49%) (Supplemental Fig. S2*C*). In IUGR female vessels treated with IBMX, the response to DEA/NO was quite similar between HUV and HUAs (Supplemental Fig. S2*D*).

In male umbilical vessels precontracted with 5-HT, AGA HUV and HUAs showed a similar relaxant response to DEA/NO (Supplemental Fig. S2*E*). In IUGR males, DEA/NO-induced relaxation was slightly but significantly lower in HUV than HUAs, with no significant difference in AUC (Supplemental Fig. S*F*). In AGA male vascular rings treated with IBMX, HUV responded significantly better to DEA/NO than HUAs but only from 3 × 10^−5^ M DEA/NO, with however no significant difference in AUC (Supplemental Fig. S2*G*). In IUGR males, HUAs and HUV showed similar DEA/NO-induced relaxation in the presence of IBMX (Supplemental Fig. S2*H*).

In female umbilical vessels preconstricted with U46619, DEA/NO-induced relaxation in AGA HUAs was slightly but significantly weaker than in HUV, with a significantly lower AUC (−18%) (Supplemental Fig. S3*A*). In IUGR females, HUV relaxed more than HUAs but only from 3 × 10^−5^ M DEA/NO, although AUC was not significantly different (Supplemental Fig. S3*B*). In AGA female vessels incubated with IBMX, DEA/NO-induced relaxation was much greater in HUV than HUAs, with a significantly higher AUC (+49%) (Supplemental Fig. S3*C*). In IUGR female vessels treated with IBMX, DEA/NO-induced relaxation was significantly greater in HUV than HUAs, with a higher AUC (+37%) (Supplemental Fig. S3*D*).

In male vascular rings preconstricted with U46619, DEA/NO-induced relaxation was significantly better in HUV than HUAs in AGA males, with a significantly higher AUC (+28%) (Supplemental Fig. S3*E*). In IUGR males, the relaxant response to DEA/NO was slightly but significantly better in HUV than HUAs, without however significant difference in AUC (Supplemental Fig. S3*F*). In the presence of IBMX, the response to DEA/NO was stronger in HUV than HUAs, both in AGA (Supplemental Fig. S3*G*) or IUGR males (Supplemental Fig. S3*H*), with significantly higher AUC (+44–45%).

RT was significantly lower in HUV than in HUAs in all groups and conditions (Table 3). The treatment with IBMX significantly reduced RT in vascular rings from all groups but to a greater extent in HUV (−59% to −75%, with 5-HT; −50% to −73% with U46619) than in HUAs (−33% to −48%, with 5-HT; −31% to −58% with U46619) (Table 3). No significant difference was found between RT measured in AGA and IUGR vessels.

Residual tension (RDT) achieved after the addition of 5-HT or U46619 was significantly higher in HUV than HUAs in all groups and conditions (Table 4). Preincubation with IBMX did not influence RDT in umbilical vessels contracted with 5-HT (Table 4). In contrast, in vascular rings constricted with U46619, IBMX significantly reduced RDT only in HUAs from IUGR males, whereas in HUV RDT was reduced by IBMX in all groups, although the observed decrease was significant only in AGA females and males (Table 4). In males, RDT was significantly lower in IUGR than AGA HUV contracted with 5-HT, independently of IBMX, as well as in IUGR HUV contracted with U46619 without IBMX compared to AGA HUV (Table 4).

**Table 4.** Residual tension achieved in vascular rings after contraction with 5HT or U46619

	F AGA	F IUGR	M AGA	M IUGR	F AGA	F IUGR	M AGA	M IUGR
	−IBMX				+IBMX			
5-HT								
HUA								
* n* (patients)	51	36	50	37	24	18	24	14
* *RDT, g	1.85 ± 0.08	2.09 ± 0.07	2.06 ± 0.09	2.07 ± 0.08	2.07 ± 0.10	1.86 ± 0.12	1.94 ± 0.08	1.80 ± 0.09
* P* value IUGR vs. AGA		0.0574		0.7666		0.2275		0.2632
* P* value +IBMX vs. −IBMX					0.1398	0.1119	0.4266	0.1012
HUV								
* n* (patients)	51	36	50	37	24	18	24	14
* *RDT, g	4.18 ± 0.16	4.04 ± 0.21	4.02 ± 0.14	3.42 ± 0.19	4.12 ± 0.23	3.56 ± 0.27	3.90 ± 0.21	3.13 ± 0.25
* P* value IUGR vs. AGA		0.4039		0.0186* (−15%)		0.1506		0.0132* (−20%)
* P* value +IBMX vs. −IBMX					0.9528	0.3029	0.2710	0.2298
* P* value HUV vs. HUA	<0.0001***	<0.0001***	<0.0001***	<0.0001***	<0.0001***	<0.0001***	<0.0001***	0.0001***
U46619								
HUA								
* n* (patients)	51	36	50	37	24	18	24	14
* *RDT, g	1.84 ± 0.07	2.01 ± 0.09	2.05 ± 0.12	2.06 ± 0.09	1.76 ± 0.09	1.92 ± 0.12	1.83 ± 0.08	1.66 ± 0.05
* P* value IUGR vs. AGA		0.1564		0.2668		0.2920		0.2204
* P* value +IBMX vs. −IBMX					0.5328	0.6461	0.7158	0.0011** (−19%)
* P* value HUA U46619 vs. 5-HT	0.7857	0.2026	0.2500	0.8858	0.0147* (−15%)	0.3750	0.1453	0.3575
HUV								
* n* (patients)	50	36	50	37	24	18	24	14
* *RDT, g	4.00 ± 0.22	4.06 ± 0.24	4.00 ± 0.16	3.28 ± 0.20†	3.16 ± 0.21	3.34 ± 0.24	3.19 ± 0.16	2.63 ± 0.25
* P* value IUGR vs. AGA		0.6553		0.0158* (−18%)		0.5500		0.1011
* P* value +IBMX vs. −IBMX					0.0176* (−21%)	0.1298 (−18%)	0.0016** (−20%)	0.0683 (−20%)
* P* value HUV vs. HUA	<0.0001***	<0.0001***	<0.0001***	0.0002***	<0.0001***	<0.0001***	<0.0001***	0.0090**
* P* value HUV U46619 vs. 5-HT	0.1935	0.6553	0.4911	0.1961	<0.0001*** (−23%)	0.0898	0.0002*** (−18%)	0.1040

Data are expressed as means ± SE. Values in parentheses correspond to the percentage of change between both groups, when difference was significant. The residual tension (RDT) corresponded to the tension achieved in the vascular ring just before addition of 2-(*N*,*N*-diethylamino)-diazenolate-2-oxide (DEA/NO). *P* values resulted from statistical analyses using a Mann-Whitney test to compare intrauterine growth restriction (IUGR) and appropriate for gestational age (AGA) or RDT in vascular rings treated or not with IBMX; a Wilcoxon matched-pairs sign rank test was used to compare human umbilical artery (HUA) and human umbilical vein (HUV) or RDT after contraction with U46619 or 5-HT. †Significant difference between males (M) and females (F) using a Mann-Whitney test. *P < 0.05, **P < 0.001, ***P < 0.0001, significant values.

The contraction induced by 100 mM potassium chloride (KCl) at the beginning of the pharmacological experiments was significantly higher in HUV than HUAs, in all groups (Table 5). No significant difference was found between AGA and IUGR females, whereas in males the contractile response induced in IUGR vessels was significantly lower than in AGA vessels, both in HUV and HUAs.

**Table 5.** Contraction induced by 100 mM KCl in HUAs and HUV

	Females		Males	
	AGA	IUGR	AGA	IUGR
*n* (patients)	75	54	74	51
HUA change in tension, g	2.13 ± 0.05	2.05 ± 0.05	2.22 ± 0.06	2.00 ± 0.06
*P* value IUGR vs. AGA		0.1952		0.0373* (−11%)
HUV change in tension, g	3.19 ± 0.12	2.96 ± 0.14	3.11 ± 0.10	2.46 ± 0.12†
*P* value IUGR vs. AGA		0.2790		0.0002*** (−21%)
*P* value HUV vs. HUA	<0.0001*** (+50%)	<0.0001*** (+44%)	<0.0001*** (+40%)	0.0017** (+23%)

Data are expressed as means ± SE of the change in tension evoked by KCl. Values into parentheses correspond to the percentage of change between both groups, for each significant difference. Contraction was induced by 100 mM KCl in human umbilical arteries (HUAs) and human umbilical vein (HUV) after equilibration of the vascular rings at the beginning of each isolated vessel tension experiment. *P* values resulted from statistical analysis using a Mann-Whitney test to compare intrauterine growth restriction (IUGR) and appropriate for gestational age (AGA) or a Wilcoxon matched-pairs sign rank test to compare HUAs and HUV. †Significant difference between males and females using a Mann-Whitney test. *P < 0.05, **P < 0.001, ***P < 0.0001, significant values.

### Western Blotting Analyses

**Figure 4.**
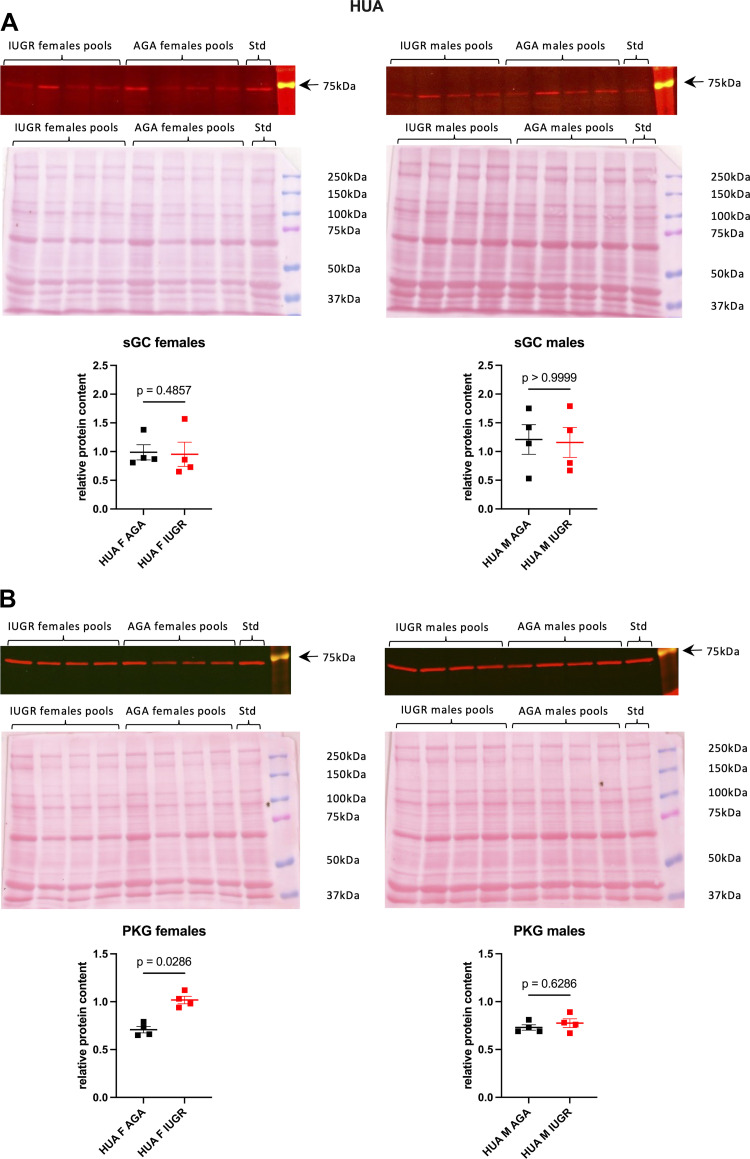
Relative protein amounts for soluble guanylyl cyclase (sGC) and cGMP-dependent protein kinase (PKG) in human umbilical arteries. F, females; M, males. Relative protein content of sGC (*A*) or PKG (*B*) was evaluated by Western blot in human umbilical artery (HUA) homogenates of appropriate for gestational age (AGA) and intrauterine growth restriction (IUGR) newborns. For each experimental group, samples from 40 patients were randomly distributed into 4 pools. For each pool, the target protein amount was normalized to the corresponding Ponceau S total protein staining and reported to the amount measured in a “standard” sample. Data are presented as individual values, with line at means ± SE (*n* = 4 pools of 10 patients). *P* values were obtained using a Mann-Whitney test to compare AGA and IUGR neonates. A representative Western blot is shown at *top*.

Two proteins implicated in the NO/cGMP relaxing pathway, sGC and PKG, were investigated by Western blot in HUA homogenates (Fig. 4). The sGC relative protein content was similar between HUA from AGA and IUGR females or males (Fig. 4*A*). The PKG relative protein amount was significantly higher in HUAs from IUGR than AGA females, whereas it was similar between AGA and IUGR males (Fig. 4*B*).

**Figure 5.**
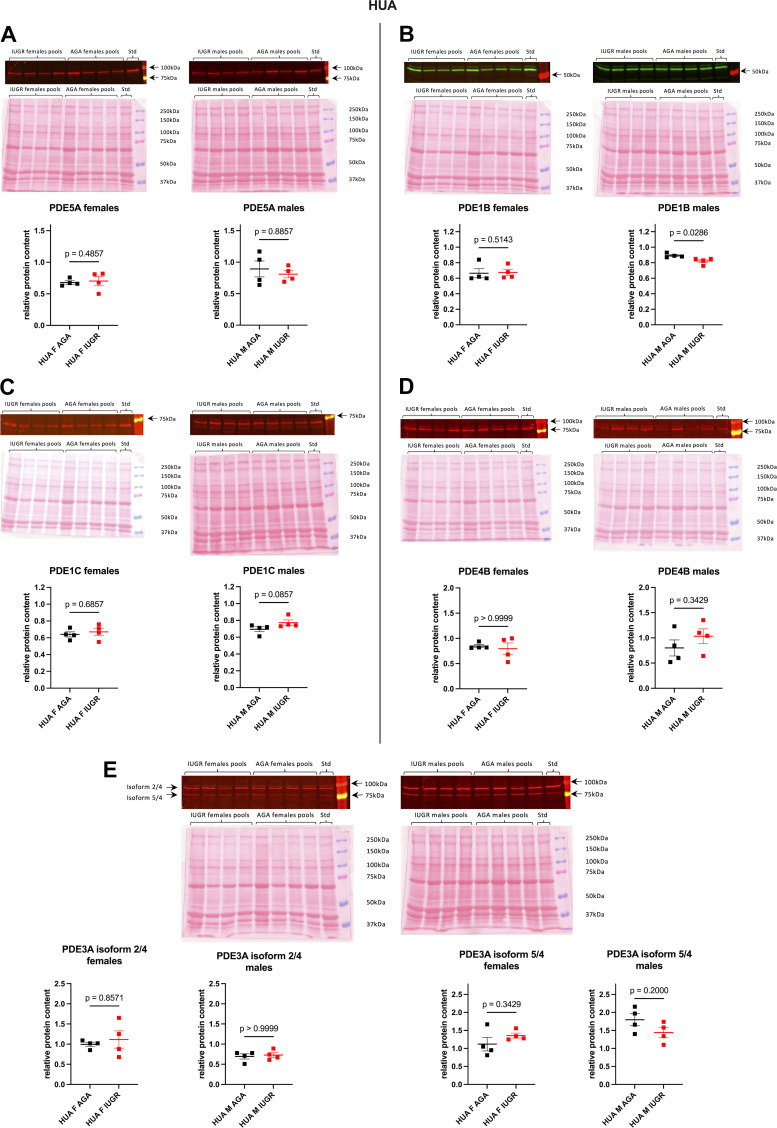
Relative protein amounts for several IBMX-sensitive phosphodiesterase (PDE) isoforms in human umbilical arteries. F, females; M, males. Relative protein content of PDE5A (*A*), PDE1B (*B*), PDE1C (*C*), PDE4B (*D*), and PDE3A (*E*) was evaluated by Western blot in human umbilical artery (HUA) homogenates of appropriate for gestational age (AGA) and intrauterine growth restriction (IUGR) newborns. For each experimental group, samples from 40 patients were randomly distributed into 4 pools. For each pool, the target protein amount was normalized to the corresponding Ponceau S total protein staining and reported to the amount measured in a “standard” sample. Data are presented as individual values, with line at means ± SE (*n* = 4 pools of 10 patients). *P* values were obtained using a Mann-Whitney test to compare AGA and IUGR neonates. A representative Western blot is shown at *top*.

Several IBMX-sensitive PDE isoforms, PDE5A, PDE1B, PDE1C, PDE3A, and PDE4B, were also investigated in umbilical vessel homogenates (Fig. 5 and Supplemental Fig. S4). For PDE3A, two bands can be observed on the membranes between 75 kDa and 100 kDa (Fig. 5*E* and Supplemental Fig. S4*E*); according to the manufacturer's indications, the lower one could correspond to the PDE3A isoform 5 (83 kDa) or isoform 4 (87 kDa) (therefore called “PDE3A isoform 5/4” in the present report) and the higher one to the PDE3A isoform 2 (91 kDa) or isoform 4 (87 kDa) (then called “PDE3A isoform 2/4” in this report). In HUA, the relative protein content for all these PDEs was similar between AGA and IUGR females (Fig. 5). In males, no significant difference was found in relative protein amount for PDE5A, PDE1C, PDE4B, and PDE3A between AGA and IUGR newborns (Fig. 5*A* and *C*–*E*), whereas a significant although slight decrease in PDE1B protein content was observed in IUGR males (Fig. 5*B*). In HUV, the relative protein content was similar between AGA and IUGR newborns for all these PDE isoforms (Supplemental Fig. S4).

Supplemental Figs. S5 and S6 show, for each studied protein, the comparison of their relative amount between HUA and HUV. For sGC, no significant difference was found between HUV and HUA, either in females (Supplemental Fig. S5*A*) or in males (Supplemental Fig. S5*B*). For PKG, the relative protein content was similar between both vessels in AGA females but higher in HUA than HUV in IUGR females, although the difference was not statistically significant (*P* = 0.0571) (Supplemental Fig. S5*C*). In males, PKG protein amount was similar between HUV and HUAs (Supplemental Fig. S5*D*).

Regarding PDEs, the relative amount of almost all studied PDE isoforms was significantly greater in HUAs than HUV, in all groups, the few others being similar between the two vessels (Supplemental Fig. S6).

### Phosphodiesterase Activity

Total and IBMX-sensitive PDE activity was assessed in HUV homogenates by quantifying the degradation of cGMP or cAMP (Table 6). No significant difference was found between AGA and IUGR newborns or between males and females. IBMX-sensitive degradation of cGMP was significantly lower than total cGMP hydrolysis in each group and represented ∼32–37% of the total cGMP PDE activity. IBMX-sensitive hydrolysis of cAMP was significantly lower than total cAMP inactivation in all groups and accounted only for ∼15–17% of the total cAMP PDE activity. Total cAMP hydrolysis was significantly higher than total cGMP hydrolysis in each group. In contrast, IBMX-sensitive cAMP degradation was significantly lower than IBMX-sensitive cGMP hydrolysis in AGA and IUGR females, whereas no significant difference was found in males.

**Table 6.** Cyclic nucleotide phosphodiesterase activity in HUV homogenates

	Females		Males	
	AGA	IUGR	AGA	IUGR
*n* (patients)	8	5	8	4
Total cGMP degradation,^a^ nmol cGMP/mg protein/min	42.39 ± 3.07	41.78 ± 5.04	36.57 ± 3.57	36.93 ± 2.67
IBMX-sensitive cGMP degradation,^a^ nmol cGMP/mg protein/min	14.89 ± 1.40*** (35%)	16.00 ± 1.34* (38%)	11.53 ± 1.21*** (32%)	13.72 ± 1.66** (37%)
Total cAMP degradation, nmol cGMP/mg protein/min	63.79 ± 2.47†††	65.47 ± 5.52†	59.73 ± 3.31††	64.14 ± 5.07†
IBMX-sensitive cAMP degradation, nmol cGMP/mg protein/min	9.42 ± 1.17***† (15%)	9.14 ± 0.81**† (14%)	9.52 ± 0.95*** (16%)	10.59 ± 1.73** (17%)

Data represent means ± SE of the quantity of cGMP or cAMP degraded, expressed as nmol cyclic nucleotide/mg protein/min. cGMP degradation and cAMP degradation were measured in human umbilical vein (HUV) homogenates. Values in parentheses correspond to the percentage of IBMX-sensitive activity compared to total phosphodiesterase (PDE) activity for cGMP or cAMP. Statistical analyses were performed using Brown-Forsythe and Welch ANOVA tests. *Significant difference between IBMX-sensitive and total cyclic nucleotide PDE activity. †Significant difference between cAMP degradation and cGMP degradation. No significant difference was found between intrauterine growth restriction (IUGR) and appropriate for gestational age (AGA) or between males and females. *†*P* < 0.05, **††*P* < 0.001, ***†††*P* < 0.0001, significant values. ^a^Total and IBMX-sensitive cGMP degradation data were previously published in Ref. 5 (Supplementary Table S3). Reused with permission from Elsevier © 2020.

## DISCUSSION

In the present study, we demonstrated that NO-induced relaxation is not impaired in HUAs of growth-restricted newborns, in contrast to HUV whose relaxant response to NO is reduced in growth-restricted compared to AGA females, whereas no significant difference was found in males (5).

More importantly, this study showed that the effects of PDE inhibition by IBMX depend on the presence of IUGR, the sex of the newborn, the vessel type, and the vasoconstrictors used to precontract the vascular rings, as summarized in Table 7. Such observations could be of particular interest for the development of therapeutic interventions using PDE inhibitors.

**Table 7.** Summary of the effects of phosphodiesterase inhibition by IBMX on human umbilical arteries and vein precontracted with 5-HT or U46619

	HUAs				HUV			
Effect of IBMX	♀ AGA	♀ IUGR	♂ AGA	♂ IUGR	♀ AGA	♀ IUGR	♂ AGA	♂ IUGR
Precontraction with 5-HT								
RT	↓	↓	↓	↓	↓↓	↓↓	↓↓	↓↓
RDT	–	–	–	–	–	–	–	–
DEA/NO-induced relaxation	–	↑	–	–	↑	↑↑	↑	–
Precontraction with U46619								
RT	↓	↓	↓	↓	↓↓	↓↓	↓↓	↓↓
RDT	–	–	–	↓	↓	(↓)	↓	(↓)
DEA/NO-induced relaxation	↑	↑	↑	↑	↑↑	↑↑	↑↑	↑↑

Summary of the effects of the nonspecific phosphodiesterase inhibitor IBMX on the vasoreactivity of isolated human umbilical arteries (HUAs) or human umbilical vein (HUV) vascular rings preconstricted with 5-HT or U46619. For each vasoconstrictor, the resting tension (RT; corresponds to the lowest tension achieved by vascular rings), the residual tension [RDT; corresponds to the tension achieved following contraction with 5-HT or U46619, measured at the time the first dose of 2-(*N*,*N*-diethylamino)-diazenolate-2-oxide (DEA/NO) was added], and the DEA/NO-induced relaxation were either increased (↑), decreased (↓), or not influenced (–) by the presence of IBMX, depending on the study group, the vessel type, and the vasoconstrictor used to precontract the vascular rings. AGA, appropriate for gestational age; IUGR, growth-restricted newborns.

### Effect of PDE Inhibition on the Human Umbilical Vasoreactivity

Our isolated vessel tension experiments showed that, in umbilical vessels pretreated with IBMX, the RT achieved in all groups and conditions was significantly lower than in the absence of this inhibitor, proving that IBMX-sensitive PDEs contribute to the vascular tone homeostasis in HUAs and HUV.

Both vasoconstrictors used to precontract the umbilical vascular rings before addition of cumulative doses of DEA/NO act on vascular smooth muscle cells via binding to specific G protein-coupled receptors, inducing IP_3_ production, cytosolic calcium concentration increase, and myosin light-chain kinase (MLCK) activation, leading to contraction through its action on muscle fibers (15–17). However, the vasoconstriction induced by the TxA2 analog U46619 was shown to involve additional mechanisms, including stimulation of some PDEs, as observed in the human saphenous vein (18) and in rat carotid arteries (19). 5-HT and U46619 were chosen to precontract the vascular rings in our experiments because preliminary data showed that they were able to induce a sustained vasoconstriction in HUV and HUAs, thus providing adequate conditions to study DEA/NO-induced relaxation (6). Moreover, both 5-HT and TxA2 have been detected in the human umbilical circulation and play important roles (20, 21).

In our study, preincubation with IBMX did not influence the RDT achieved in umbilical vessels contracted by 5-HT, demonstrating that 5-HT-induced contraction is independent of activation of IBMX-sensitive PDEs. In contrast, when umbilical vessels were constricted with U46619, preincubation with IBMX reduced RDT achieved in HUV from all groups, although the reduction was statistically significant only in AGA females and males; in HUAs, IBMX significantly reduced RDT only in IUGR males. These data suggest that, in those vessels, the inhibition of IBMX-sensitive PDEs promotes a dilatory response counterbalancing the contraction induced by U46619, probably because U46619 stimulates some PDEs. This is consistent with the observations made in human saphenous vein (18) and rat carotid arteries (19). The differences between data obtained in umbilical vascular rings precontracted with 5-HT or U46619 could therefore be related to the activation of some PDEs by U46619.

In umbilical vessels precontracted with U46619, preincubation with IBMX significantly improved DEA/NO-induced relaxation in both HUAs and HUV (5) in all study groups. In contrast, in umbilical vessels preconstricted with 5-HT, the relaxant response of HUAs to DEA/NO was not influenced by pretreatment with IBMX, except in IUGR females, where IBMX enhanced NO-induced relaxation, whereas in HUV IBMX significantly improved DEA/NO-induced relaxation in all groups except in IUGR males (5). These results indicate that the effects of IBMX on NO-induced relaxation are influenced by the presence of IUGR, the sex of the newborn, the type of umbilical vessel, and the vasoconstrictor involved.

### sGC, PKG, and PDEs

To explain the various pharmacological responses described above, we assessed the relative content of several proteins implicated in the NO/cGMP signaling pathway in HUAs and HUV. sGC and PKG were studied as prorelaxant components of the NO/cGMP relaxing pathway, whereas several IBMX-sensitive PDE isoforms (PDE1B, PDE1C, PDE3A, PDE4B, and PDE5A) were investigated as counteracting partners. These PDE isoforms belong to PDE families known to contribute to the regulation of vascular smooth muscle relaxation (22, 23), which have been previously detected in human umbilical vessels (24). PDE5A hydrolyzes specifically cGMP, whereas PDE4B selectively degrades cAMP. The other isoforms studied are dual PDEs hydrolyzing cGMP and cAMP: PDE1B, degrading preferentially cGMP, and PDE1C, inactivating both cGMP and cAMP, are stimulated by binding of the calcium-calmodulin complex, following a raise in cytosolic calcium concentration; finally, PDE3A is a cGMP-inhibited cAMP-selective PDE, because it hydrolyzes cAMP 10 times faster than cGMP but has a higher affinity for cGMP, which is therefore a competitive inhibitor of cAMP degradation (22, 23).

In males, the relative protein content observed for sGC, PKG, and the different PDE isoforms was similar between AGA and IUGR HUA, except for a slight decrease in PDE1B in IUGR versus AGA HUA. In HUV, no significant difference was found between AGA and IUGR males for sGC, PKG (5), and each PDE isoform studied. These results are consistent with the similar relaxant responses to DEA/NO observed in both groups in HUAs and HUV (5), suggesting that the NO/cGMP signaling pathway is not impaired in human umbilical vessels of growth-restricted newborn males.

In females, sGC protein amount in HUA was similar in both groups, which is consistent with the similar relaxant response to DEA/NO observed in IUGR and AGA female HUAs. In contrast, PKG protein abundance was significantly higher in IUGR than AGA female HUA, which could be a compensatory mechanism counterbalancing some alterations in IUGR females, thus resulting in similar NO-induced relaxation in both groups. We previously showed, in HUV, a significant increase in sGC protein amount, accompanied by a higher cGMP production, in IUGR compared to AGA females, as well as an increase in PKG protein content in IUGR females (5). Similar results were obtained in the present study by repeating these Western blotting analyses under the same conditions as for HUA (data not shown) to allow comparison between both vessel types. The observed increase in sGC and PKG protein content is surprising given the decrease in NO-induced relaxation found in IUGR compared to AGA female HUV and probably reflects compensatory mechanisms attempting to counteract the overall impairment of umbilical vasorelaxation observed in IUGR female HUV.

It is interesting to note that PKG protein content is increased in both HUAs and HUV from IUGR females. It would be therefore of interest to investigate whether this trend could be found in other vessels of these individuals. Such an increase in PKG, if it persists throughout life, could be of potential benefit in some treatments to improve vasorelaxation in case of cardiovascular disorders later in life.

Regarding PDEs, no significant difference was found for PDE1B, PDE1C, PDE3A, PDE4B, and PDE5A between AGA and IUGR female umbilical vessels, but it cannot be excluded that a differential expression could occur for other isoforms. Nevertheless, our investigations were limited by the large number of PDE isoforms (more than 100 isoforms known in humans to date; Ref. 23) and the lack of adequate specific antibodies; we, therefore, focused on the main IBMX-sensitive isoforms known to be involved in the regulation of vascular tone. To circumvent these technical limitations, we decided to assess the total PDE activity and hypothesized that their overall activity might be increased in IUGR HUV compared to AGA. However, we did not measure any significant difference in total or IBMX-sensitive cGMP degradation between AGA and IUGR HUV, either in females or in males. A similar observation was done for cAMP degradation. Our data showed that IBMX-sensitive PDE activity accounted for only 32–37% of the total hydrolyzing activity for cGMP and 15–17% for cAMP. It could be therefore relevant to investigate IBMX-insensitive PDE isoforms in HUV and HUAs, like PDE8 and PDE9, two IBMX-insensitive isoforms expressed in vascular smooth muscle cells (23).

Interestingly, the cGMP and cAMP degradation measured in HUV homogenates was in the nanomolar range, while the NO-induced production of cGMP we previously found in HUV rings was in the picomolar range (5). Such observation suggests that the measurement of PDE activity in homogenates may not reflect what happens in the native tissue. Indeed, cGMP and cAMP degradation has been measured, for technical reasons, in vessel homogenates, leading to a loss of tissular and intracellular organization and thus of the resulting regulation, whereas the cGMP production has been determined in intact vascular rings, in the same conditions as for pharmacological studies (5). Our data argue in favor of the crucial role of tissue organization, cell-cell interactions, and probably also subcellular organization. Indeed, there is increasing evidence that subcellular microdomains and signalosomes (macromolecular signaling complexes) play a key role in the regulation of physiological processes (25). Such compartmentation appears to be particularly important in cyclic nucleotide signaling in vascular smooth muscle cells (23). Because of the lack of relevance of such experimental conditions, we did not repeat the experiments to measure the PDE activity in HUA homogenates.

Therefore, neither specific relative protein contents nor enzymatic activities data provided a direct explanation for the changes in vasoreactivity observed between IUGR and AGA females, suggesting the involvement of other proteins and/or the contribution of the subcellular organization to the regulation of umbilical vascular tone under physiological or pathological conditions. In female HUV, the impaired NO-induced relaxation observed in the absence of IBMX in IUGR compared to AGA, despite increased sGC and PKG protein contents, could result from the counteracting of their activity by an elevated PDE activity or from alterations in the downstream signaling cascade. In HUAs, the NO-induced relaxation was similar in AGA and IUGR females in the absence of IBMX, despite the significant increase in PKG protein amount found in IUGR compared to AGA females, probably also because of high PDE activity or alterations in PKG effectors. The greater sGC and/or PKG protein contents however could explain the improved response to NO observed in IUGR female vessels treated with IBMX. This argues in favor of an impairment of cGMP signaling by a high PDE activity in IUGR females but does not exclude other alterations in the downstream cascade. The beneficial effects of IBMX on the NO-induced relaxation in HUV and HUAs from IUGR females suggest a subcellular colocalization of sGC, PKG, and IBMX-sensitive PDEs in these vessels.

### 5-HT versus U46619

The relaxant response to DEA/NO was significantly better when HUAs and HUV were precontracted with U46619 rather than 5-HT, in all groups and conditions, suggesting that U46619 improves the response to NO in both vessels, even in the absence of IBMX. Such data suggest that the PDEs stimulated by U46619 probably do not directly interact with DEA/NO-induced cGMP production, either because they hydrolyze only or preferentially cAMP or because of distinct subcellular localization. Another explanation could be related to the inhibition of multidrug resistance protein 4 (MRP4) by U46619. Indeed, the membrane transporters MRP4 and MRP5 were found to be able to export cGMP and cAMP (26). cGMP export appeared to play a key role in the regulation of vascular relaxation, similar to enzymatic cGMP degradation (27). As the transport by MRP4 was shown to be strongly inhibited by prostaglandins and thromboxane B2 (an inactive metabolite of TxA2) (28), it is likely that the TxA2 analog U46619 could also inhibit cyclic nucleotide export by MRP4, thus reducing cGMP and cAMP efflux, resulting in an increased NO-induced relaxation in HUV and HUAs. There is growing evidence that MRP4 could be a potential target for cardiovascular disease (29): MRP4 inhibition was able to prevent human coronary and pulmonary arteries smooth muscle cell proliferation, as well as pulmonary hypertension in mice (30); moreover, an upregulation of MRP4 was found in pathological conditions, like in pulmonary arteries from patients with pulmonary arterial hypertension or in mice exposed to hypoxic conditions. Recently, inhibition of MRP4 in corpus cavernosa from obese mice was found to increase cGMP accumulation in smooth muscle cells, thus improving the relaxation of smooth muscle and erectile function in these mice (31). Further investigation of MRP4 in HUV and HUAs would therefore be of interest.

In all groups, preincubation of HUAs and HUV with IBMX resulted in a change in the curve shape of the relaxant response to DEA/NO when vascular rings were precontracted with U46619, but not with 5-HT, especially in HUV, suggesting activation of another mechanism leading to vasorelaxation. Given that U46619 was found, in rat carotid arteries, to stimulate PDE3 and PDE4 (19), both IBMX-sensitive cAMP degrading PDEs, it is likely that their inhibition by IBMX could lead to the enhancement of cAMP-mediated relaxation. The change in curve shape could therefore result either from the addition of cGMP- and cAMP-mediated relaxation or from the potentiation of NO-induced vasodilation by the activation of the cAMP-mediated relaxation pathway. Indeed, there is a crosstalk between cGMP and cAMP signaling pathways (23).

In females, in HUV precontracted with 5-HT, the presence of IBMX completely abolished the impairment of NO-induced relaxation observed in IUGR compared to AGA females (5), suggesting a strong implication of IBMX-sensitive PDEs in this alteration. In contrast, in HUV precontracted with U46619, the relaxant response to DEA/NO was improved by IBMX in both groups but remained significantly weaker in IUGR than AGA females. This could be due to the activation of IBMX-insensitive PDEs by U46619 in IUGR female HUV. Such a hypothesis would be consistent with the significant increase in RT observed in HUV from IUGR females precontracted with U46619 compared to 5-HT, in the absence or presence of IBMX, whereas no significant difference was found in the other groups. As suggested above, investigation of PDE8 and PDE9 could therefore be relevant, given the great IBMX-insensitive PDE activity found in HUV homogenates. PDE9 appears as a good candidate in IUGR female HUV because it hydrolyzes cGMP, whereas PDE8 hydrolyzes cAMP. Interestingly, in rat aortic smooth muscle cells, PDE1, PDE5, and PDE9 were found to cooperate in the local control of cGMP concentration (32).

In HUAs precontracted with 5HT, IBMX did not change the relaxant response to DEA/NO in AGA and IUGR males, as well as in AGA females, suggesting that IBMX-sensitive PDEs did not contribute to the regulation of NO-induced relaxation in these vessels. In IUGR female HUAs, however, the addition of IBMX significantly improved the relaxant response to NO, suggesting the contribution of a high IBMX-sensitive cGMP degrading PDE activity, counterbalancing the elevated PKG protein content. In contrast, when HUAs were precontracted with U46619, IBMX significantly improved NO-induced relaxation in all groups, probably through inhibition of IBMX-sensitive PDEs activated by U46619, thus enhancing cAMP-mediated relaxation. It was however surprising that, in female HUAs precontracted with U46619, the relaxant response to DEA/NO in the presence of IBMX was similar between IUGR and AGA females, rather than higher in IUGR females as expected based on the improvement observed in IUGR female HUAs and the absence of effect on AGA female HUAs when these vessels were precontracted by 5-HT. That could be due to the activation of an IBMX-insensitive cGMP hydrolyzing PDE by U46619 in IUGR female HUAs, such as PDE9, as suspected in IUGR female HUV.

### HUV versus HUAs

Taken together, these findings led us to wonder whether a treatment using PDE inhibitors would really benefit the fetus. One could argue that, given the lack of alteration in NO-induced relaxation observed in HUAs, it does not matter whether the treatment applied to improve HUV relaxation has less beneficial effects on HUAs, as long as it does not induce adverse effects. However, we suspect that the potential resulting imbalance between umbilical venous and arterial blood flow could have deleterious effects on the fetus. Indeed, it is likely that a greater increase in blood flow in the HUV than in the HUAs could possibly induce adverse effects. We therefore decided to directly compare the vasoreactivity of HUV to that of HUAs.

Our isolated vessel tension experiments showed that RT was significantly lower in HUV than HUAs, in all groups and conditions. This is not surprising given that veins are generally considered to be “simple conduits,” whereas arteries are more muscularized vessels with a higher blood pressure. Based on our previous histomorphometric measurements on umbilical cord sections (6), the total cross-sectional area was higher in HUV than HUAs, whereas the percentage of smooth muscle in total cross-sectional area was indeed lower in HUV (78–80%) than HUAs (93–98%) (see Supplemental Table S3).

However, after stimulation by 5-HT or U46619, the RDT achieved in HUV was significantly higher than in HUAs, in all groups and conditions. Similarly, the contraction induced by 100 mM KCl was significantly greater in HUV than in HUAs. These observations suggest that, despite a thinner and less muscularized vascular wall, HUV has a great vasoactive potential and could therefore play a key role in the regulation of the blood flow from the placenta to the fetus. Because 5-HT, U46619, and KCl induce vasoconstriction through an elevation of cytosolic calcium concentration leading to activation of MLCK and modification of muscle fibers, the higher vasoconstrictive potential of HUV could be related to a larger intracellular calcium pool, a greater ability to release calcium from the endoplasmic reticulum, higher MLCK activity, or differences in muscle fibers compared to HUAs. Investigation of proteins constituting the cytoskeleton could help to understand the different vasoconstrictive properties of HUV and HUAs. In IUGR male HUV, the RDT achieved after contraction with 5-HT or U46619 was lower than in AGA males. Likewise, the vasoconstriction induced by 100 mM KCl was significantly weaker in IUGR than in AGA males in HUV and HUAs. This could reflect alterations in the IP_3_-MLCK signaling pathway or muscle fibers in IUGR males.

The addition of IBMX resulted in a significant decrease in RT in all vessels, which was stronger in HUV than HUAs, suggesting a greater contribution of IBMX-sensitive PDEs to vascular tone homeostasis in HUV than HUAs. This is surprising given that, for all PDE isoforms studied, the relative content was either higher in HUAs than HUV or similar between the two vessels. Because all PDE isoforms investigated in this report were IBMX sensitive, the greater effect of IBMX on HUV despite a lower relative content in PDEs could be due either to the fact that 10^−4^ M IBMX was not sufficient to efficiently block all these PDEs in HUAs or, more likely, that there is an “uncoupling” between some PDEs and the NO/cGMP relaxing pathway in HUAs, related to their respective subcellular localization.

A direct comparison of the relaxant response to DEA/NO between HUV and HUAs from each patient showed that, overall, IBMX enhanced NO-induced relaxation to a greater extent in HUV than HUAs, leading to a potential imbalance between these two vessels. It is likely that such an imbalance in blood flow between HUV and HUAs could have adverse effects on fetal hemodynamics.

Overall, the greater increase in NO-induced relaxation observed in the presence of IBMX in HUV than in HUAs, despite a higher IBMX-sensitive PDEs protein content in HUAs, suggested that subcellular compartmentation could play an important role in the regulation of the umbilical vascular tone. This subcellular organization seems to differ between HUV and HUAs, allowing a differential control of interactions between cGMP and cAMP signaling pathways. The existence of cGMP compartmentation was previously described in HUA smooth muscle cells, where the particulate pool of cGMP, synthesized near the membrane by the particulate guanylyl cyclase (pGC), was controlled by PDE3 and PDE5, whereas cytosolic cGMP, generated by sGC, was controlled exclusively by PDE3 (33). Our data suggest however that subcellular compartmentation could vary depending on the type of vessel, the sex of the newborn, and the presence of IUGR.

Taken together, our data show that, in our experimental conditions, the HUV has a greater reactivity to pharmacological agents than HUAs. Moreover, the relative amount found for the various proteins studied could help to partially explain the pharmacological reactivity of umbilical vessels but was not sufficient to directly account for the differences observed between HUV and HUAs and between AGA and IUGR newborns. Therefore, subcellular compartmentation appears to play an important role in the reactivity of HUAs and HUV and needs to be further investigated to better understand the regulation of umbilical vascular tone in physiological and pathological conditions.

There are few studies directly comparing the pharmacological reactivity of HUV and HUAs. Moreover, most were performed in physiological solution aerated with 95% O_2_, instead of low oxygen level as in the present project, and several used a segment of umbilical vessel near the placenta, whereas this study was focused on the fetal part of the umbilical cord. However, the vasoreactivity of umbilical vessels is known to vary depending on the oxygen level or the location along the umbilical cord (20, 21, 34). In an organ bath bubbled with 2.5% O_2_, HUV and HUAs from the middle part of the umbilical cord showed similar contraction in response to 10^−8^ M U46619 or 60 mM KCl and were only partially relaxed by the nonspecific PDE inhibitor papaverine when contracted with KCl, but showed a maximal relaxation when constricted with U46619 (35). In an organ bath aerated with 95% O_2_, HUV and HUAs isolated from the placental part of the cord in term newborns, showed similar contraction to U46619 10^−6^ M and relaxant response to forskolin or sodium nitroprusside, whereas relaxation induced by the sGC stimulator BAY41-2272 was greater in HUV than HUAs; furthermore, the PDE3 inhibitor milrinone and PDE4 inhibitor rolipram were more efficient than sildenafil to relax HUAs and HUV precontracted with U46619 (36), which is consistent with the assumption that U46619 stimulates PDE3 and PDE4 in both vessels. It should however be noted that, in the latter study, U46619-induced contraction was expressed as a percentage of the contraction evoked by 62.5 mM KCl in each vessel, but it was not mentioned whether the response to KCl was similar in HUV and HUAs or not. Finally, most studies did not consider the sex of the donor. Comparison between the results of the present report and previously published data is therefore difficult due to variations in experimental approaches.

### Limitations

There are some limitations to our study. The dose responses to DEA/NO in the absence or presence of IBMX have been established in two separate sets of patients. We first studied the NO-induced relaxation in umbilical vessels, and then, based on the results, we decided to test the effects of IBMX. However, we previously demonstrated in HUV that assessment of DEA/NO-induced relaxation with or without IBMX in the same patients led to similar conclusions (5). Moreover, we checked that both subsets of patients have the same demographic characteristics. In contrast, HUV and HUAs have been studied simultaneously during each organ bath experiment, the results of which are presented in this report and in our previous publication (5), thus allowing direct comparison between HUV and HUAs vasoreactivity using matched-paired statistical analyses.

Regarding DEA/NO, the pharmacological experiments should have been started with lower concentrations: in fact, in the presence of IBMX, the first dose has already a nonnegligible effect since IBMX increases the sensitivity to NO. Nevertheless, this does not undermine the overall conclusions of this study.

Our study focused on the smooth muscle part of the NO/cGMP pathway. The contribution of the endothelium to the regulation of umbilical vascular tone thus remains to be assessed in that context and will certainly add complexity to the regulatory mechanisms highlighted here.

Extrapolation of our observations to the in vivo situation must be done with caution. Indeed, this study was based on the use of the nonspecific PDE inhibitor IBMX, whereas in vivo more specific agents are usually administered to avoid or limit side effects. These experiments should therefore be repeated using other PDE inhibitors, like sildenafil, milrinone, rolipram, or the PDE9 inhibitor BAY73-6691 (22, 37). In addition, it is not known to what extent the drug can cross the placental barrier. In any case, it seems likely that the concentration of this substance will be lower in the HUAs than in the HUV, further reducing its effect, which seems already attenuated in the HUAs. Another issue regarding the translation of our results to the in vivo situation is that the effects of PDE inhibition depend on the vasoconstrictor acting on the vessels. Both 5-HT and TxA2 have been detected in the human umbilical circulation (20, 21). Their concentrations vary depending on gestational age, nutritional status, and the presence of some maternal or fetal pathologies, like preeclampsia or IUGR where high levels of 5-HT and TxA2 have been found (38–40). The impact of a treatment using PDE inhibitors would therefore depend on many factors.

### PDE Inhibition in Clinical Trials

Although based on the use of the nonspecific PDE inhibitor IBMX rather than sildenafil, our findings may provide some insights into the lack of benefits observed in the STRIDER study: as mentioned in the introduction, this international randomized placebo-controlled trial failed to show any beneficial effect of a maternal sildenafil treatment in severe early-onset fetal growth restriction on survival, perinatal morbidity, pregnancy duration, fetal growth velocity or birthweight (7, 8, 41). An increased rate of PPHN in the treated group was even found in the Dutch STRIDER arm (7, 9). The authors hypothesized post hoc that PPHN could be due to a “rebound” vasoconstriction following cessation of the treatment (9). Based on the data of the present study, another potential explanation could be that sildenafil treatment could lead to some imbalance in the umbilical circulation by promoting relaxation in HUV more than in HUAs, thus resulting in unexpected adverse effects on the fetal hemodynamics.

These observations could be useful for further reflection about the administration of PDE inhibitors: although these drugs are widely used to treat several pathologies without major side effects, any new indication should be preceded by extensive in vitro investigations to evaluate potential beneficial and side effects on the whole targeted system. Both physiological and pathological conditions should be considered, as well as the sex of the patient.

### Perspectives

**Figure 6.**
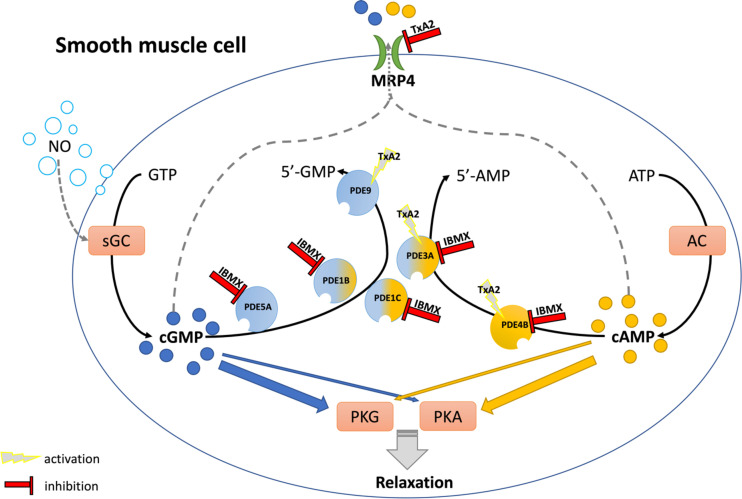
Interactions contributing to the nitric oxide/cyclic guanosine monophosphate (NO/cGMP)-mediated relaxation in human umbilical vascular smooth muscle cells. This illustration summarizes the complex interactions implicated in the nitric oxide-induced relaxation of the umbilical vascular smooth muscle cells, as suggested based on the results of the present study. Further investigations, using notably more specific/selective phosphodiesterase (PDE) inhibitors, need to be performed to confirm our hypotheses. 5′-AMP, 5′-adenosine monophosphate; 5′-GMP, 5′-guanosine monophosphate; ATP, adenosine triphosphate; AC, adenylyl cyclase; cAMP, cyclic adenosine monohosphate; PKG, cGMP-dependent protein kinase; GTP, guanosine triphosphate; IBMX, 3-isobutyl-1-methylxanthine; MRP4, multidrug resistance-associated protein 4; PKA, cAMP-dependent protein kinase; sGC, soluble guanylyl cyclase; TxA2, thromboxane A2.

Figure 6 summarizes the complex interactions implicated in the NO-induced relaxation of the umbilical vascular SMCs, as suggested based on the results of the present study. Further investigations, using notably more specific/selective PDE inhibitors, need to be performed to confirm our hypotheses. Moreover, this figure does not include the contribution of subcellular microdomains, which probably vary between HUV and HUAs.

To complete the present study, there is a need to further investigate the contribution of subcellular compartmentation to the regulation of the human umbilical vascular tone. Additional functional studies should assess endothelium-dependent relaxation but also the effects of natriuretic peptides, whose receptors are membrane-bound proteins with guanylyl cyclase activity (referred to as pGC). Because of different subcellular localizations, sGC and pGC probably interact with distinct PDE pools, thus allowing specific regulation of cGMP signaling. Such investigations may provide a better understanding of how to improve fetoplacental perfusion while maintaining a balance between blood flow in the HUV and HUAs. Other activators of the cGMP-mediated relaxation should also be considered, such as sGC activators and stimulators (42).

### Conclusions

In conclusion, despite a thinner and less muscularized vascular wall, the HUV shows a greater reactivity to pharmacological agents than the HUAs. Moreover, the effects of PDE inhibition vary depending on the presence of IUGR, fetal sex, vessel type, and vasoconstrictors acting on the vessels. This finding draws attention to the need for caution in the development of therapeutic interventions based on the use of PDE inhibitors to improve the placental-fetal circulation. The regulation of the umbilical vascular tone is complex and depends not only on the relative content and activity of the different proteins implicated but also on the subcellular organization, which mediates protein interactions. More broadly, our findings add to the growing evidence supporting the need to consider sex as an important biological variable in cardiovascular research.

Further investigations are therefore needed to better understand the regulation of the human umbilical circulation in physiological and pathological conditions and the role of subcellular microdomains. This may allow the design of effective therapeutic strategies to prevent or limit the development of IUGR and its short- and long-term consequences.

## DATA AVAILABILITY

Data will be made available upon reasonable request to the corresponding author.

## SUPPLEMENTAL DATA

10.5281/zenodo.10828902 Supplemental Figs. S1–S6 and Supplemental Tables S1–S3: https://doi.org/10.5281/zenodo.11192217.

## GRANTS

This work was supported by the Swiss National Science Foundation Grant 32003B_138491 and the W. and E. Grand d'Hauteville Foundation for Academic Biomedical and Nursing Research.

## DISCLOSURES

No conflicts of interest, financial or otherwise, are declared by the authors.

## AUTHOR CONTRIBUTIONS

A-C.P. and J-F.T. conceived and designed research; M.B., F.D., and S.M. performed experiments; S.J. collected patient data; A-C.P., M.B., and S.M. analyzed data; A-C.P. interpreted results of experiments; A-C.P. and S.M. prepared figures; A-C.P. drafted manuscript; A-C.P., F.D., S.J., S.M., D.B., and J-F.T. edited and revised manuscript; A-C.P., M.B., F.D., S.J., S.M., D.B., and J-F.T. approved final version of manuscript.
